# Wrap-like transfer printing for three-dimensional curvy electronics

**DOI:** 10.1126/sciadv.adi0357

**Published:** 2023-07-26

**Authors:** Xingye Chen, Wei Jian, Zhijian Wang, Jun Ai, Yu Kang, Pengcheng Sun, Zhouheng Wang, Yinji Ma, Heling Wang, Ying Chen, Xue Feng

**Affiliations:** ^1^ Laboratory of Flexible Electronics Technology, Tsinghua University, Beijing 100084, China.; ^2^ Institute of Flexible Electronics Technology of THU, Zhejiang, Jiaxing 314000, China.; ^3^ AML, Department of Engineering Mechanics, Tsinghua University, Beijing 100084, China.; ^4^ School of Materials Science and Engineering, Tsinghua University, Beijing 100084, China.; ^5^ Qiantang Science and Technology Innovation Center, Hangzhou 310016, China.

## Abstract

Three-dimensional (3D) curvy electronics has wide-ranging application in biomedical health care, soft machine, and high-density curved imager. Limited by material properties, complex procedures, and coverage ability of existing fabrication techniques, the development of high-performance 3D curvy electronics remains challenging. Here, we propose an automated wrap-like transfer printing prototype for fabricating 3D curvy electronics. Assisted by a gentle and uniform pressure field, the prefabricated planar circuits on the petal-like stamp are integrated onto the target surface intactly with full coverage. The driving pressure for the wrapping is provided by the strain recovery of a prestrained elastic film triggered by the air pressure control. The wrapping configuration and strain distribution of the stamp are simulated by finite element analysis, and the pattern and thickness of the stamps are optimized. Demonstration of this strategy including spherical meander antenna, spherical light-emitting diode array, and spherical solar cell array illustrates its feasibility in the development of complex 3D curvy electronics.

## Teaser

A wrap-like transfer printing strategy is used to integrate electronics onto 3D curvy surfaces automatically with full coverage.

## INTRODUCTION

Three-dimensional (3D) curvy surfaces are ubiquitous in living organisms, industrial equipment, and natural environment. Relying on integrating different material systems and functional units onto 3D curvy surfaces intimately, 3D curvy electronics starts to gain momentum in biomedical health care (*1*–*3*), structural health monitoring (*4*), reprogrammable metasurfaces (*5*, *6*), and soft machines (*7*, *8*). Compared with their planar counterparts, 3D curvy electronics has better performance owing to the advantages of spatial device arrangement, excellent curvilinear conformability, large integration area, etc. For instance, a high-density curved imager array can enable wide-field and lower aberration with simpler lens systems (*9*–*12*). 3D electrically small antennas (ESAs) have relatively wider bandwidth and higher efficiency compared with those in planar designs (*13*–*15*). Moreover, the electronics is required to be conformally contacted with the 3D shape of biological tissue (e.g., brain, heart, and cornea), providing health monitoring or medical treatment capabilities (*16*–*18*). 3D curvy electronics has overcome the performance choke point of planar electronic devices to a certain extent and is expected to promote the continued development of Moore’s law (*19*, *20*).

Up to now, several techniques have been attempted in achieving 3D curvy electronics (*21*), including 3D printing, holographic lithography, kirigami/origami assembling, and transfer printing. 3D printing enables almost any shape of electronics using particular conductor and semiconductor inks (*22*–*25*). However, the material property, such as conductivity and carrier mobility, fails to be as effective as those in wafer-based technologies, which inevitably impairs the performance of printed 3D electronics (*26*, *27*). Holographic lithography makes use of light interference to create patterns on curvilinear surfaces. Bihelical tracks have been fabricated onto a conical substrate, exhibiting a bright prospect of the 3D patterning approach. However, the mask must be designed by complex computation, rendering its quite rare application (*28*). Kirigami assembling with self-healing materials is used to fabricate 3D conformal electronics manually, and the conductivity of interconnects is slightly decreased after healing (*29*). Similarly, by using the origami strategy, planar electronic devices can be folded from a 2D sheet to a 3D framework (*30*–*32*). However, smooth contour, which is quite critical in particular applications such as contact lens, is impossible to achieve by this approach because of the nonexistence of infinitely many infinitesimally small folds.

Transfer printing is another efficient approach to achieve 3D electronic devices, in which the stamps are adopted to pick up devices from the donor substrates and print them onto the receiver substrates (*33*). At the outset, most transfer printing methods are only applicable for planar devices (*34*, *35*). So far, several transfer printing technologies have been developed for 3D electronic devices with nondevelopable surface, which can be divided into two strategies, i.e., direct transfer printing and transfer printing before shaping. In the direct transfer printing process, to conform with curvy surfaces, low elastic modulus materials are desirable for the stamp, similar to inflated elastomeric balloon (*36*), or even liquid droplet (*37*). However, the electronics can only be conformal to a localized portion of the target surface by this approach. In the other strategy, the 3D hemisphere substrate is prefabricated with elastomers and flattened with biaxial tension. Then, the picked-up device is printed onto the strained elastomer substrate and transforms into a 3D hemisphere shape after releasing the prestrain (*38*). This strategy relies on a radially stretched substrate as the transfer medium and adopts compressible interconnects (e.g., island-bridge structure) to accommodate the large mechanical strain, resulting in impairing the integration density of functional components. Taking advantage of the high resolution of wafer-based technologies, transfer printing is quite appealing in fabricating 3D electronics and in developing a generic transfer printing strategy for 3D curvy electronics, with full coverage of the target surface remaining highly desired.

Here, we propose a manufacturing technique called wrap-like transfer printing for fabricating 3D curvy electronics reliably. The approach can ensure planar electronics to fully wrap the target curvilinear surfaces by the petal-like elastomer stamp. The prefabricated planar electronics is picked up by the petal-like stamp and printed onto the adhesive-treated target sphere automatically assisted by a gentle and uniform pressure field. A homemade prototype tool is adopted to provide the driving force for the wrap-like transfer printing. The pattern and thickness of the stamps are optimized through the wrapping configuration and strain distribution analysis by finite element analysis (FEA). Furthermore, several 3D curvy electronics [spherical meander antenna, spherical light-emitting diode (LED) array, spherical solar cell array, etc.] are fabricated by the wrap-like transfer printing, indicating its feasibility and reliability.

## RESULTS

### Wrap-like transfer printing

**Fig. 1.**
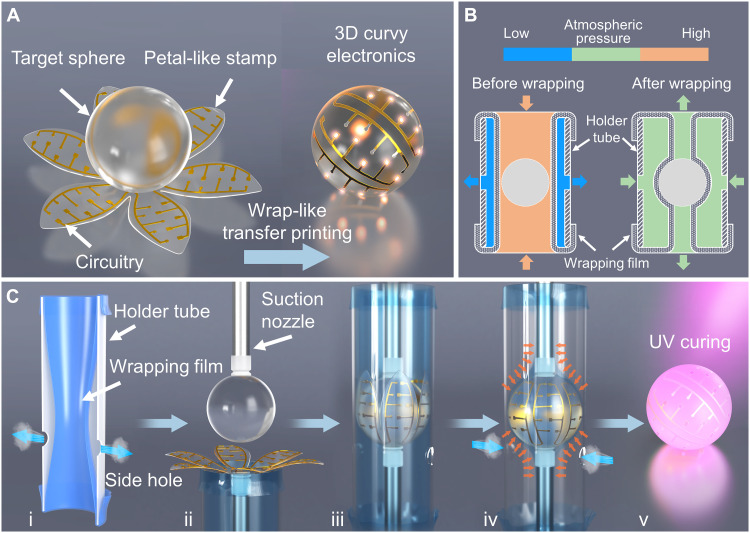
The wrap-like transfer printing process. (**A**) Schematic illustration of wrap-like transfer printing relied on a petal-like stamp with circuitry. (**B**) Schematic diagram of pressure distribution before and after wrapping (arrows represent the direction of airflow). (**C**) The fabrication process includes the following: (i) inflate the wrapping film. (ii) Align the target sphere and the petal-like stamp. (iii) Insert the sphere and petal-like stamp into the wrapping film. (iv) Contract the wrapping film and print the circuitry onto the target sphere automatically. (v) Cure the adhesives between the circuitry and the sphere.

The wrap-like transfer printing strategy proposed here is inspired by packaging the Mozartkugel (a spherically shaped sweet) with a golden foil for efficient mass production (*39*). Before transfer printing, the circuitry is fabricated by the mature planar techniques, and the chips are die-bonded and wire-bonded on the circuitry, forming the planar electronics. With the assistance of the petal-like stamp (composite of a rubber elastomer and water-soluble adhesive tape), the planar electronics is picked up and printed onto the target 3D surface intactly with full coverage, as shown in Fig. 1A. The fabrication details are described in Materials and Methods and fig. S1.

The key to the conformability of transfer printing is the delicate control of the wrapping process. A homemade prototype tool is developed for wrap-like transfer printing. In addition, the automatic and gentle wrapping is realized with the assistance of the strain recovery of a prestrained elastic film triggered by air pressure control. As illustrated in Fig. 1B, the prototype is composed of a hyperelastic wrapping film (latex rubber) and a rigid holder tube (polymethyl methacrylate). Moreover, there are two suction nozzles temporarily holding the planar electronic device and the 3D target sphere. The wrapping film is the inner bladder of the holder tube, and it is fixed and sealed at both ends. In preparation, high-pressure air is injected into the wrapping film, making the film inflate circumferentially and forcing the air between the holder tube and the wrapping film to escape through the side holes. Then, the side holes are covered to block the airflow. In this way, the air pressure in the wrapping film is higher than that between the holder tube and the wrapping film (approximate vacuum state), shown in the left of Fig. 1B. The pressure difference keeps the wrapping film stuck on the tube wall (Fig. 1C, i). At the moment, the mechanical equilibrium of wrapping film is governed by internal atmospheric pressure, prestretch load (induced by sealing procedure at both ends), and tensile stress in the inflated wrapping film (fig. S2B). Then, the two suction nozzles are aligned and used to hold the target sphere and the petal-like stamp correspondingly (Fig. 1C, ii). Note that the cross section of the holder tube is narrower than the planar size of the petal-like stamp, so it is reasonable that the petals are half-wrapped on the sphere in the inserting process (Fig. 1C, iii). When the target moves to the right place, the side holes are unlocked. The existing pressure difference drives the air to flow into the space between the holder and the wrapping film. The air pressure on both sides of the film eventually evolves to the standard atmospheric pressure (Fig. 1B, right). The original mechanical equilibrium cannot be maintained for the barometric disturbance; the new equilibrium state of wrapping film is governed by external atmospheric pressure, internal atmospheric pressure, prestretch load, and updated tensile stress in the wrapping film (fig. S2D). Attributed to the disappearance of air pressure difference, the pretensioned wrapping film tends to recover to its uninflated state. In this contracting process of the wrapping film, the petal-like stamp is driven to wrap the target sphere entirely by a gentle and uniform pressure field, as shown in Fig. 1C (iv) and movie S1. Benefitting from the excellent conformability of the petal-like stamp, the electronics is printed onto a 3D curvy surface intactly with full coverage capability. Note that the target surface is coated with adhesives in advance; after the adhesives are cured by ultraviolet (UV) illumination or another curing method, the circuitry is firmly integrated with the 3D profile, as shown in the right of Fig. 1C. Ultimately, the stamp is removed by dissolving the water-soluble tape, indicating the finish of the process.

### Petal-like stamp geometric design and wrapping strategy optimization

**Fig. 2.**
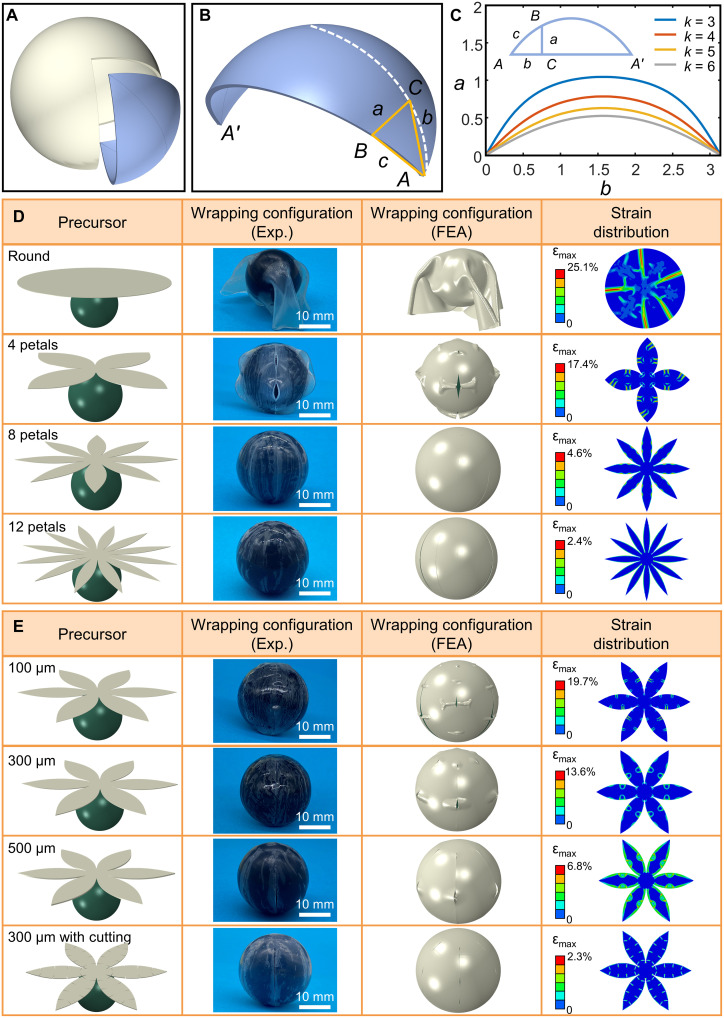
The geometry design of a petal-like stamp and optimization of the wrapping strategy. (**A**) Disassemble schematic of a spherical surface. (**B**) Labeled petal-like stamp. (**C**) Comparison of stamp contour with different petal numbers. (**D**) Experimental (Exp.) images and finite element analysis (FEA) prediction of wrapping strategies with different petal numbers (stamp thickness is 200 μm for each strategy). (**E**) Experimental images and FEA prediction of wrapping strategies with different stamp thicknesses and specific cutting treatment (six petals for each strategy).

If simply only adopting an elastic round sheet to wrap the sphere, then undesirable distortions and wrinkles will be generated inevitably in the transfer printing process (*40*). Therefore, it is necessary to design the planar geometry of stamps, which plays a crucial role in the conformability of transfer printing. Now, consider cutting a 3D sphere into *k* pieces through its great circular arc, from its south pole to north pole, *k* identical petal-like cells will be obtained (for simplicity, a cell of four-petal stamp is shown in Fig. 2A). Provided that the planar geometry of the stamp corresponds to the flattened pattern of the petal-like cell, the stamp will fit the spherical surface after wrapping.

To determine the 2D geometry of the petal-like stamp, the following mathematical analysis is carried out. Focusing on the petal-like stamp wrapping the unit sphere, the half petal angle *A* can then be determined to be π/*k* according to spherical trigonometry. The point *C* is specified on the great circular arc, *b* is the distance traveling from point *C* to south pole *A*, and *a* is the length of the line drawn perpendicular to the arc and intersected with the edge of the petal (Fig. 2B and the inset of Fig. 2C). The 2D contour of the petal-like stamp is obtained by the value of *a* in terms of *b*, which can be given by $a(b)=\arctan\left(\sin b\tan\frac{\pi }{k}\right)$ (*39*). The perimeter of the great circular arc *AA*′ is π for the unit sphere. By calculating the value of *a* in terms of *b* ∈ [0, π], the planar geometry of *k*-petal stamp is obtained, as presented in Fig. 2C. The results suggest that the stamp profile becomes gentler with the increase of petal numbers. It is obvious that the single cell area of the petal-like stamp decreases with increasing petal numbers (fig. S3). Although the petal-like stamp is capable of wrapping 3D sphere exactly in geometry, the corresponding 2D pattern has gaps between the adjacent petals, which is similar to the petal-like structure of the flatten retina (*41*).

With the above discussion, the optimization of the wrapping strategy can be guided by FEA. The petal number, pattern, and thickness of the stamp are the main parameters affecting the transfer printing quality. For the case of a round stamp (thickness is 200 μm), to correspond to the great circular arc of the target sphere, the diameter of the round stamp is designed as 2π*R*, where *R* is the radius of the target sphere. Uniform pressure load is applied to the upper surface of the stamp (the pressure direction is always perpendicular to the surface) in the wrapping process (fig. S9). As mentioned above, severe wrinkles and overlaps are observed after the wrapping process, and the stamp does not fit tightly to the sphere surface with interspace. The maximum principal strain (ɛ_max_) contour of the stamp after wrapping is shown in Fig. 2D (top). The corresponding maximum ɛ_max_ in the stamp is 25.1%, which is far larger than the fracture strain of the metal circuitry (~8% for Cu) (*42*). For the case of petal-like stamp wrapping, the results show that the petal will suffer the compressive stress, which is inevitable when using a flat piece to wrap an undevelopable curvy surface (fig. S4). Wrinkles will be induced by compressive stress on stamps and circuits, which results in nonideal conformability of the stamp with receiver surface and even hurting the electronics.

Figure 2D provides experimental images and FEA prediction of wrapping configuration and strain distribution of wrapping strategies with different petal numbers (the stamp thickness is 200 μm for each strategy). The results indicate that, with increasing the petal number, the stamp wrapping morphology becomes smoother and the number of wrinkles is reduced. When the petal number reaches 8 or more, a wrinkle-free wrapping can be guaranteed. Moreover, the strain concentration in the stamp is most evident in the regions of near petal outline. With the increase of petal number, the maximum ɛ_max_ in the stamp gets weakened and the area of strain concentration is declined. However, the petal number cannot be increased indefinitely. If so, then the petal-like stamp will be shattered into smaller pieces, which causes inconvenience in circuit layout and diminishes the degree of circuit integration. In general, a six-petal stamp is able to meet the quality requirement of wrap-like transfer printing.

The generation of wrinkles can be regarded as a result of mechanical instability of the stamp under the compressive stress. This structural instability can be prevented by increasing stamp thickness. Figure 2E displays experimental images and FEA prediction of wrapping configuration and strain distribution of wrapping strategies with different petal thicknesses (six-petal stamp for each strategy). It is obvious that, as the stamp thickness increases, wrinkling becomes less noticeable and the maximum ɛ_max_ in the stamp is reduced. However, the flexibility of the stamp is reduced with the increase of thickness, because the bending stiffness *EI* can be markedly increased by *EI* ∝ *h*^3^, where *E*, *I*, and *h* represents the elastic modules, moment of inertia, and thickness of the stamp, respectively. The 500 μm petal-like stamp is an appropriate choice, trading off wrinkles and flexibility. Furthermore, the cutting treatment is attempted on the basis of the petal-like design (fig. S5). Through cutting notches at the edge of the petal, the mechanical constraint of the stamp is relieved and the accumulated strain energy in the wrapping process can be released. The notches will serve as artificial structure defects that absorb compressive strain and prevent wrinkle formation. For the six-petal stamp with a thickness of 300 μm, the result shows that the wrapping configuration is perfect and the maximum ɛ_max_ in the stamp with cutting treatment can be reduced to 2.3% compared with no cutting method (13.6%), as shown in Fig. 2E (bottom).

In the actual wrap-like transfer printing experiments, it is found that a six-petal stamp with a thickness of 500 μm can already provide a good-enough conformability of transfer printing. Therefore, the following wrap-like transfer printing demonstrations and applications are all based on a six-petal stamp with a thickness of 500 μm. Various 2D patterns are printed to the sphere surface by wrap-like transfer printing (fig. S6). Furthermore, integrating diverse electrodes on an expandable balloon through wrap-like transfer printing is carried out, showing a good conformability (fig. S7). Circular dot array and serpentine electrodes are also printed on contact lenses successfully (fig. S8). The results show that the wrap-like transfer printing process also works for other 3D curvy surfaces (e.g., ellipsoid and corneal shape), not just sphere, exhibiting good engineering versatility.

To study the reproducibility and precision in position control of wrap-like transfer printing, the parallel and meridian pattern is designed and then transfer printed onto the sphere (note S1 and fig. S10). The transfer printed 3D pattern is overlapped with the theoretical 3D models. The error of the position control is defined as the distance between the parallel and meridian cross points in experiments (marked in red) and the theoretical model (marked in white), respectively. As can be seen in fig. S11, the maximum position error is likely located near the north pole where the petal stamp tips meet each other. The average maximum position error in the three repeated transferred samples is 1.5 mm, which is 4.7% to the semiperimeter.

### Spherical meander antenna

The miniaturization of electronic devices and antennas is in demand for applications such as biomedical health monitoring (*43*). However, for an antenna to perform signal reception and transmission, minimizing the size directly modifies the working characteristics including operating frequency, bandwidth, radiation efficiency, and so on. The 3D layout design can compensate the shortcomings of smaller size to some degree by making full use of the limited volume space. For example, 3D ESAs offer better performance including a relatively wide bandwidth and high radiation efficiency, while planar ESAs generally have limited bandwidth and efficiency because of their small volume occupation of Chu sphere (*44*, *45*). Here, wrap-like transfer printing is carried out to fabricate a six-arm spherical meander monopole antenna.

**Fig. 3.**
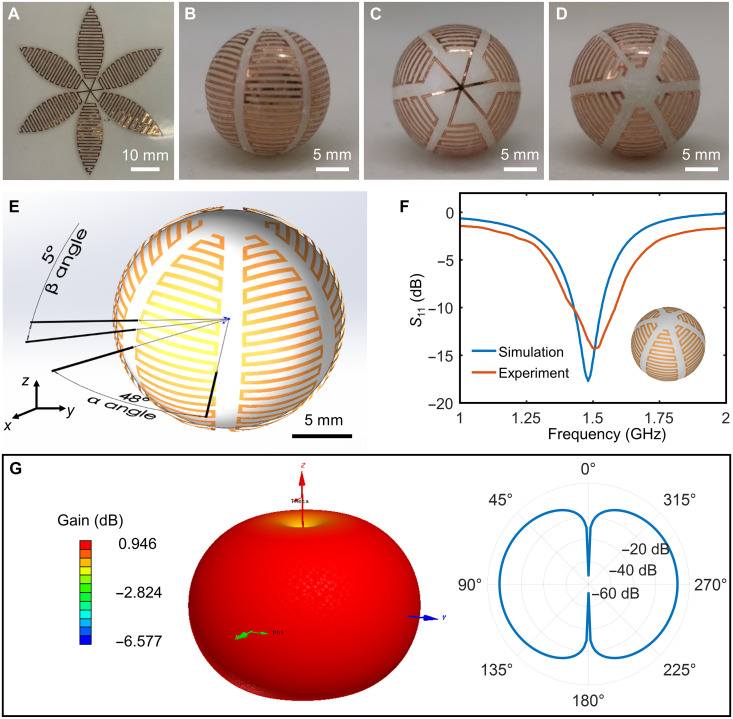
Spherical meander antenna fabricated by wrap-like printing. (**A**) An optical image of the corresponding 2D petal pattern of the spherical meander antenna. (**B** to **D**) Optical images of the spherical meander antenna after wrap-like printing (front view, bottom view, and top view, respectively). (**E**) The structure parameters of the designed spherical meander antenna. (**F**) Experimental measurement and numerical simulation of the reflection coefficient *S*_11_ of the spherical meander antenna. (**G**) Numerical simulation of 3D radiation pattern (left) and θ-sweep radiation pattern (right) of spherical meander antenna.

The antenna is prefabricated in its planar form by laser cutting copper foil (30 μm) and then picked up by the petal-like stamp. The fabrication details are described in Materials and Methods. Figure 3A shows the corresponding 2D petal pattern of the spherical meander antenna. Adopting wrap-like transfer printing, the antenna is printed onto a plastic ball (polyformaldehyde) successfully and fits well with the spherical surface, as shown in Fig. 3 (B to D). The antenna consists of multiple folded spherical meander arms connected to the south pole of the ball. According to a previous study on hemispherical meander antenna (*46*), four design parameters affect the antenna performance, including the extend angle of a single arm in *XY* plane (α angle) (Fig. 3E), the spherical angle between each parallel meander section to the *XY* plane (β angle) (Fig. 3E), the width of antenna line, and the number of arms. As a key indicator of the antenna, reflection coefficient (*S*_11_) describes how much the electromagnetic wave is reflected by an impedance discontinuity in the transmission medium, which is equal to the ratio of the amplitude of the reflected wave to the incident wave. A low reflection coefficient indicates the antenna radiates most of the power with high efficiency. The bandwidth describes the frequency range over which the antenna can transform energy effectively (threshold limit *S*_11_ < −10 dB), and the fraction bandwidth is determined by the bandwidth divided by the operating frequency. According to the simulation results (Fig. 3F), the full spherical meander antenna has a fractional bandwidth of 7.4% (bandwidth is 0.11 GHz) at an operating frequency of 1.48 GHz, when α = 48°, β = 5°, linewidth = 400 μm, and arm number = 6, in good agreement with the experimental data (operating frequency is 1.51 GHz, bandwidth is 0.16 GHz, and fractional bandwidth is 10.6%). The optimization of design parameters of the spherical meander antenna has been carried out. The results show that, under the same arm number and α, the reflection coefficient *S*_11_ improves and the operating frequency goes higher with the increase of linewidth and β. (fig. S12). The radiation patterns of the spherical antenna are obtained by the simulation, as presented in Fig. 3G. Because the spherical antenna structure is axisymmetric, the result of 3D radiation pattern exhibits a pronounced symmetry circumferentially (Fig. 3G, left). For each axisymmetric section, the θ sweep radiation pattern is shown in the right of Fig. 3G, implying that the antenna achieves a good gain in the direction perpendicular to the central axis.

### Spherical LED array

**Fig. 4.**
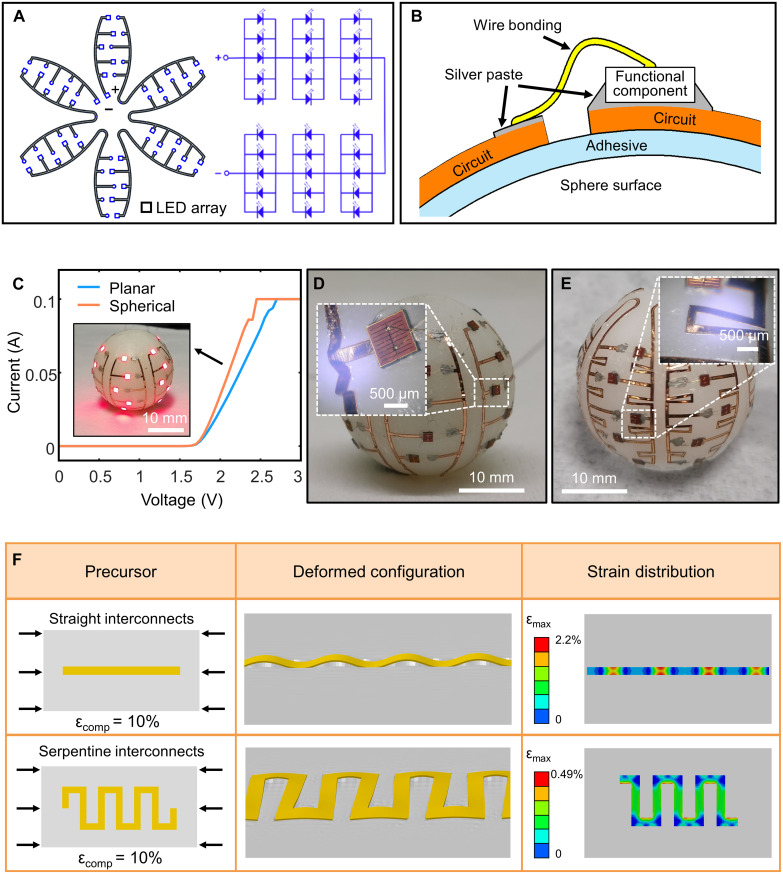
Spherical LED array fabricated by wrap-like printing. (**A**) The corresponding 2D petal-like circuitry layout and circuit schematic diagram (the square represents the position of the LED array). (**B**) Schematic of the interconnection of the functional component, attached using a silver paste and wire bonding. (**C**) *I*-*V* curves of the single LED before and after wrap-like transfer printing. Inset: Optical image of lighting LED array. (**D**) The optical image of deformed configurations for straight interconnects after wrap-like transfer printing. Inset: Optical image of local wrinkling. (**E**) The optical image of deformed configurations for serpentine interconnects after wrap-like transfer printing. Inset: Optical image of out-of-plane deformation without wrinkling. (**F**) Finite element analysis (FEA) prediction of deformation configuration and strain distribution of different interconnect designs (the applied compressive strain is 10% for each case).

To demonstrate the capability of transfer printing a completed circuit with functional components and interconnects, a spherical LED array (5 × 6; each LED is sized 1 mm by 1 mm; GaAs substrate) is fabricated as an example. The corresponding 2D petal-like circuitry layout and circuit schematic diagram is presented in Fig. 4A. In this device, 30 LEDs are connected together through a six-petal circuitry design (the square represents the position of the LED array). The interconnect circuit is first fabricated by laser cutting copper foil (30 μm), and LEDs with one-end-free wire are die-bonded at the specific bottom electrode position of the circuit using a conductive silver paste. Adhering the LED’s free-end wire to the circuit using a conductive silver paste finishes the preparation of the 2D array. The petal-like stamp then picks up the 2D LED array and prints them on to spherical surface (Fig. 4B). The fabrication details are described in Materials and Methods and fig. S1. The 360° lighting performance of the LED array is displayed as shown in movie S2. The inset of Fig. 4C shows that the LED array is lighted successfully on the spherical surface. The current-voltage relation (*I-V*) characteristics of the single LED before (on the planar substrate) and after (on the sphere) transfer printing is shown in Fig. 4C. The turn-on voltage of the LED is the same before and after the transfer printing, indicating that the LED works properly on the sphere. The results verify the reliability of wrap-like transfer printing.

Moreover, to better accommodate the mechanical strain during wrap-like transfer printing process, the interconnects, especially for those located at the edge of the petal stamp, need to be designed. Figure 4 (D and E) shows the deformed configurations of different interconnect designs after wrap-like transfer printing (the inset is the zoom-in optical image). Local wrinkling and interfacial debonding are obviously observed for straight interconnect designs (metal thickness is 30 μm and line width is 300 μm), and no wrinkling for serpentine interconnects (metal thickness is 30 μm and line width is 300 μm). As mentioned above, the petal will suffer compressive stress in the wrap-like transfer printing process. The FEA prediction of the deformed configuration and the maximum principal strain (ɛ_max_) contour of straight interconnects (the thickness is 30 μm and the line width is 300 μm) and serpentine interconnects (the thickness is 30 μm, the line width is 300 μm, the serpentine height is 1.6 mm, and the serpentine width is 1 mm) is shown in Fig. 4F and fig. S13, and the applied compressive strain is 10% for each case (the substrate thickness is 500 μm). It is clear that the out-of-plane wrinkling appears because of the mechanism of instability under compression, accompanied by the risk of interface debonding. However, the serpentine interconnects accommodate large compression via not only in-plane bending but also out-of-plane bending and twisting (*47*), without the appearance of wrinkling. Under the same compressive strain, the out-of-plane displacement of straight interconnects is higher than that of serpentine interconnects. Compared to the straight interconnects, the maximum ɛ_max_ of the serpentine interconnects can be reduced to 0.49% (2.2% for the straight interconnects), which contributes to improving the circuit reliability in the transfer printing process.

### Spherical solar cell array

**Fig. 5.**
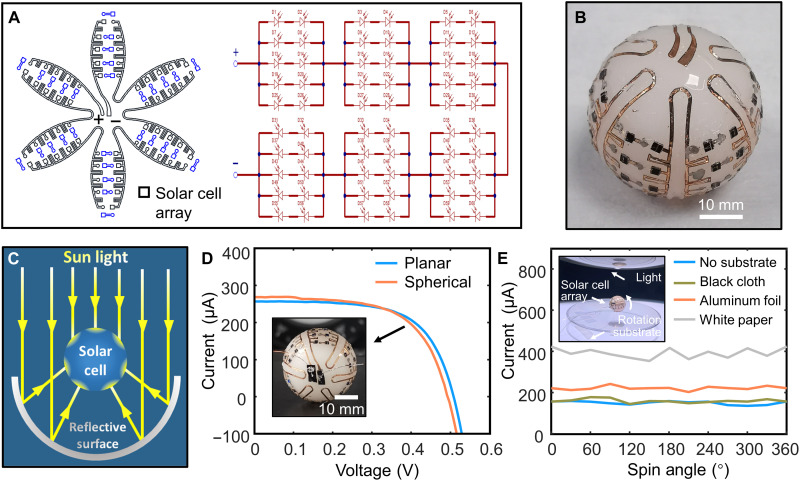
Spherical solar cell array fabricated by wrap-like printing. (**A**) The corresponding 2D petal-like circuitry layout and circuit schematic diagram. (**B**) The optical image of the spherical solar cell array after transfer printing. (**C**) Schematic of how spherical solar cell array benefits from back-reflection. (**D**) *I*-*V* curves of the cell unit before and after transfer printing. Inset: Optical image of the solar cell array. (**E**) The output current of the device under the simulated sunlight with different substrates.

Compared to a traditional flat-plate cell, a 3D spherical solar cell increases the power output with the same projection area, because a sphere can receive light three-dimensionally (*48*, *49*). Here, a spherical solar cell array (10 × 6; each cell sizes 1 mm by 1 mm) is fabricated by wrap-like transfer printing. The corresponding 2D petal-shaped circuit layout and circuit schematic are shown in Fig. 5A. Metal interconnects for the electrical connection is formed by laser cutting copper foil (thickness is 30 μm and linewidth is 300 μm). The solar cell is integrated onto the circuit in a similar way with which the LEDs are mounted as mentioned above. Then, the solar cell array is picked up and transfer printed to a spherical surface using a petal-like stamp. The detailed fabrication process is described in Materials and Methods and fig. S1. Figure 5B shows an optical image of spherical solar cell array. The device arrays in spherical form can capture light three-dimensionally and provide a steady power supply, which are advantageous in energy harvesting. It is capable of not only receiving the energy of direct light but also absorbing the light reflected by the background material, as shown in Fig. 5C.

The seraphical cell array is measured under ambient conditions of 100 mW cm^−2^ (1 Sun). The *I*-*V* response of a single cell is compared before (on the planar substrate) and after (on the sphere) transfer printing (Fig. 5D), showing that the transfer printing process has no noticeable influence on cell function. The output current of the device is tested under simulated sunlight, achieved by rotating the device around its symmetry axis (the inset of Fig. 5E). Different background substrates are also applied to monitor the effect of reflection light. The results indicate that the device provides a steady output current from a wide-angle range of light. Compared to the spherical solar cell array placed on the workbench directly (no substrate), the device is able to capture the greatest amount of reflective light with white paper, followed by aluminum foil and black cloth. This phenomenon is due to the different types of reflectance obtained in each case (*49*). The incoming light is bounced in all directions with the diffuse reflection effect of white paper. However, light is reflected in almost one direction by a mirror-like surface of aluminum foil. The diffuse reflectance of black paper is negligible, so it does not contribute to the increase of output current.

## DISCUSSION

In summary, we propose a wrap-like transfer printing strategy for manufacturing 3D curvy electronics relying on the homemade prototype. The planar electronics can be integrated on to the 3D target curvy surface intactly with full coverage by this automatic approach. Guided by the FEA results, the wrap-like transfer printing strategy is optimized in terms of petal numbers, stamp thickness, and precutting treatment. The approach is applicable for different curvilinear surfaces, including sphere, ellipsoid, and corneal shape, exhibiting good conformability of electronics. In addition, the approach is adopted to fabricate various 3D curvy electronics to illustrate engineering versatility. A spherical meander antenna is created with an operating frequency of 1.51 GHz, fractional bandwidth of 10.6%, and omnidirectional radiation pattern. An LED array with GaAs substrate is transferred onto the sphere and lighted successfully. Moreover, a spherical solar cell array is fabricated and can provide a steady power supply from the wide-angle light source. The wrap-like transfer printing strategy reported here overcomes the existing limitations of developing high-performance 3D curvy electronics. To conclude, the wrap-like transfer printing strategy reported here is featured with good compatibility, easiness to reproduce, and low cost compared with other reported methods for 3D curvy electronics (table S1). The wrap-like approach accommodates heterogeneous integration of various high-performance inorganic electronics on the 3D curvy surface, getting rid of material shortcomings (i.e., low conductivity and low carrier mobility) of particular conductor and semiconductor inks by 3D printing. The wrap-like approach is realized by a simple physical mechanism with low-cost equipment, so it is easier to reproduce than holographic lithography or 3D printing, which requires expensive equipment or sophisticated computation in design. Compared with the kirigami/origami assembling strategy, the wrap-like approach produces 3D devices in smooth profile without the folding edges. Regarding to the direct transfer printing methods with balloons or water drops as the stamps, the wrap-like approach is more superior in the full coverage and precise position.

However, there are also several aspects that should be improved further. One is that the driving pressure for the wrapping provided by the strain recovery of the prestrained elastic film can be regulated more meticulously. The geometry parameter, elastic modulus of wrapping film, and air pressure difference between the two sides of the film play a critical role in the uniform and gentle pressure. For the case of more fragile circuitry, it is necessary to apply an appropriate pressure field, preventing circuitry failure while maintaining good conformability. The other is that the flexibility and conformability of inorganic functional components can be improved. Because of the intrinsic material properties (i.e., high hardness, high brittleness, and low facture toughness) of inorganic semiconductor materials, an irreversible damage of thick chip (thickness is 160 μm in this work) may occur in the wrap-like transfer printing. Ultrathin chips (with thickness less than 50 μm) (*50*) exhibit high flexibility and excellent deformability for low bending stiffness, which can be integrated in the circuitry to enhance the conformability of the electronics.

## MATERIALS AND METHODS

### Petal-like stamp preparation

First, a plastic mold was 3D printed to the target shape using a stereolithography resin printer (Lite300HD, UnionTech, China). Second, silicone rubber components (00-30A/B, Smooth-On, USA) were poured into the plastic mold and cured at room temperature for 24 hours. Next, a piece of water-soluble double-sided adhesive tape (51915, TESA, China) was cut into exactly the same shape as the pattern of stamp by laser cutting (DirectLaser C1, DCT, China). Subsequently, the surface of the rubber petal-like stamp was treated with UV/ozone for 30 min, after which the patterened water-soluble double-sided adhesive tape was stuck to the rubber petal-like stamp. Last, the conformal petal-like stamp was ready after removing the releasing film from the tape layer.

### Spherical meander antenna fabrication

First, a 2 mm thick polydimethylsiloxane (PDMS) substrate (Sylgard 184; mixing ratio is 10:1) was attached to a flat glass plate. Second, a 30 μm thick copper foil was bonded to the PDMS/glass substrate by electrostatic bonding. Then, the copper foil was cut into the designed shape of antenna by laser cutting (DirectLaser C1, DCT, China). After that, the planar antenna was aligned and picked up by the petal-like stamp carefully. Next, the target POM (polyoxymethylene) ball was coated with a layer of UV curable adhesives (ergo 8500, Kisling, Switzerland). Subsequently, the meander antenna was transfer printed onto the POM ball by wrap-like transfer printing. Next, the device was illuminated by ultraviolet exposure (λ = 365 nm) to cure the adhesives between the electronics and the spherical surface, with a dose of 200 mJ cm^−2^ for 10 min. Last, the petal-like stamp was removed by water soaking, and the fabrication of spherical meander antenna was completed.

Multiple methods should be applied to control the stamps overlapping in the wrapping process. First, stamp with less petal number must be used as long as it provides a good-enough wrapping performance, because a wider petal has better stiffness against lateral displacement (fig. S14), which is the main reason of overlapping. Second, the inner diameter of the wrapping film and the holding tube need to be carefully chosen. The ideal diameter will be slightly smaller than the sum of ball diameter plus two times the petal thickness, which forms a tight fit when inserting the ball and petal into the tube; thus, the overlapping area will be too thick to be inserted to the tube and pushed back to where it should be by the tube edge. Third, the center of the sphere target and the center of the stamp that must be precisely aligned when entering the holder tube thus provide a good symmetrical wrapping state and prevent petals from overlapping. Last, proper lubrication must be applied to the wrapping film to free the stamp from the possible tangential force imposed by the wrapping film, leaving only normal pressure to facilitate the wrapping.

### Spherical LED (solar cell) array fabrication

First, an LED (or a solar cell) was bonded onto the PCB (printed circuit board) using a conductive silver paste, and ultrasonic wire bonding (WT2310, WETEL, China) was conducted to form a gold wire connection from an electrode on the LED to the PCB pad. Then, the bond of gold wire to the pad and the bond of LED to PCB were both debonded using a lancet, resulting in an LED unit with a hanging gold wire on its electrode. Next, a 30 μm thick copper circuit was fabricated on the PDMS/glass substrate by laser cutting (DirectLaser C1, DCT, China). After that, the previous made LED-gold wire unit was mounted onto the circuit using a conductive silver paste (05001-AB, SPI Supplies, USA) via heating to 190°C for 15 s. The completed circuit was then transferred onto the POM ball by wrap-like transfer printing. The following procedures are the same as the fabrication of a spherical meander antenna.

### Finite element analysis

FEA was performed using a commercial software package (ABAQUS, version 2018). In the FEA analysis, the geometric center of the petal-like stamp was precisely aligned with the upper vertex of the target sphere in the assembly step. The target sphere was considered stationary, and all its degrees of motion freedom (including translation and rotation) were restricted, i.e., the displacement components were set as *U_x_* = 0, *U_y_* = 0, *U_z_* = 0, *UR_x_* = 0, *UR_y_* = 0, and *UR_z_* = 0. Uniform pressure load was applied to the upper surface of the stamp. The pressure direction was always perpendicular to the surface in the wrapping process. The pressure was inactive at the end of the wrapping. Note that, in the experiment, the target surface and the petal-like stamp were bonded with water-soluble adhesives; “rough” tangential behavior and “hard” normal behavior were exploited to simulate the contact interaction between the stamp and the sphere. In addition, the surfaces remain fully bonded together once they contact; therefore, separation after contact was not allowed. The stamp was modeled as a four-node shell element (S4R) and the sphere was modeled as a discrete rigid element. A refined mesh with feature sizes smaller than 1/40 of the sphere radius was adopted to ensure accuracy. The Mooney-Rivlin hyperelastic constitutive model was adopted to capture the mechanical properties of the stamp, with the elastic modulus (*E*) and Poisson’s ratio (*v*) being *E*_stamp_ = 2.4 MPa and *v*_stamp_ = 0.49 (the relevant parameters being *C*_10_ = 0.3221 MPa, *C*_01_ = 0.0805 MPa, and *D*_1_ = 0.05 MPa^−1^). In the simulation of compressing interconnects, note that, in the existence of water-soluble adhesives between the substrate and the interconnects, the “tie” constraint was used to bond interconnects on the substrate. The displacement components were set as *U_x_* = 0 and *U_z_* = 0 for the left side of the substrate as well as *U_x_* = 0 and *U_z_* = ɛ_comp_ *L_x_* for the right side of the substrate, where ɛ_comp_ is the applied compressive strain and *L_x_* is the length of the substrate along *x* direction. The material parameters were defined as follows: *E*_Cu_ = 119 GPa and *v*_Cu_ = 0.34 for Cu as well as *E*_substrate_ = 2.4 MPa and *v*_substrate_ = 0.49 for the substrate.

### Electromagnetic simulations and antenna measurement

The commercial software ANSYS HFSS (High Frequency Structure Simulator) was adopted to conduct electromagnetic simulations of reflection coefficient, operating frequency, bandwidth, and radiation pattern of the spherical antennas. The 3D configuration of antenna was imported in the software ANSYS HFSS. The relative permittivity (ɛ_r_), relative permeability (μ_r_), and conductivity (σ) are defined as follows: ɛ_rCu_ = 1, μ_rCu_ = 0.999991, and σ_Cu_ = 5.8 × 10^7^ S m^−1^ for Cu as well as ɛ_rsubstrate_ = 3.5, μ_rsubstrate_ = 1, and σ_substrate_ = 2.5 × 10^−14^ S m^−1^ for substrate. In the experimental measurement, the antenna characteristics were measured with a vector network analyzer (E5063A, Keysight).

### Spherical LED and solar cell array measurement

The *I-V* characteristics of LED and solar cell were measured using a Keithley 2400 source meter. Simulated sunlight (100 mW cm^−2^) was provided using a solar simulator (Solia, Newport Oriel, USA). The output current from a wide-angle range of light was achieved by rotating the spherical solar cell array around its symmetry axis.

## Supplementary Materials

### This PDF file includes:

Note S1 Figs. S1 to S14 Table S1 Legends for movies S1 and S2
