# Supplementary material for: Wrap-like transfer printing for three-dimensional curvy electronics
Source: Sci Adv. 2023 Jul 26;9(30):eadi0357. doi: 10.1126/sciadv.adi0357 (PMC10371014; doi:10.1126/sciadv.adi0357)
Supplement: Supplementary file 1 — Note S1 Figs. S1 to S14 Table S1 Legends for movies S1 and S2 [file sciadv.adi0357_sm.pdf]

Science Advances
AAAS

Supplementary Materials for

Wrap-like transfer printing for three-dimensional curvy electronics

Xingye Chen *et al.*

Corresponding author: Ying Chen, chenying@ifet-tsinghua.org; Xue Feng, fengxue@tsinghua.edu.cn

Sci. Adv. **9**, eadi0357 (2023)
DOI: 10.1126/sciadv.adi0357

The PDF file includes:

Note S1
Figs. S1 to S14
Table S1
Legends for movies S1 and S2

Other Supplementary Material for this manuscript includes the following:

Movies S1 and S2

Note S1. The space mapping relation between the target 3D curve and the corresponding 2D pattern.

We have built a mathematic model to describe the space mapping relation between the 2D patterns and the 3D target. As shown in the fig. S10A, the position of the target point P on the sphere with radius R can be denoted as (R, θ, φ). Note that the target point P is located on a spherical petal, the great circular arc A_1QA_2 is the midline of the petal, the great circular arcs $A_1C_1A_2$ and $A_1C_2A_2$ are the side contour lines of the petal, and the three circular arcs are meridians of the sphere from the north pole A_1 to the south pole A_2. The parallel of the sphere passing through the point P intersects the above three great circular arcs at C_1, Q, C_2, respectively. The parallel of the sphere passing through the sphere center O intersects the above three great circular arcs at B_1, T, B_2, respectively. The spherical petal is unrolled to the planar petal presented in the fig. S10B. According to the geometric symmetry, the meridian A_1QA_2 is mapped to the straight line A_1A_2 with length of πR, the parallel B_1B_2 is mapped to the straight line B_1B_2, and the parallel C_1C_2 is mapped to the curve C_1C_2. The coordinate system is established as xoy. Based on the spherical trigonometry spherics, the 2D contour of curve A_1PA_2 is given by[39]

$$x = f(y) = R\arctan\left(\sin\left(\frac{y}{R}\right)\tan(\varphi)\right)$$

Where $y \in [0, \pi R]$ .Then the arc length of curve A_1PA_2 is obtained by

$$S(y) = \int_0^y \sqrt{1+\left(\frac{dx}{dy}\right)^2}\, dt$$

where $\dfrac{dx}{dy} = \dfrac{\tan(\varphi)\cos\left(\frac{y}{R}\right)}{1+\left(\sin\left(\frac{y}{R}\right)\tan(\varphi)\right)^2}$. Therefore, the arc length of the curve A_1PA_2 is $S(\pi R)$. Note that all the meridian of the sphere of the spherical petal have the same arc length of πR, however, the corresponding 2D patterns have the arc length larger than πR (except for the midline A_1QA_2), and the planar arc length of the meridian near the edge of the petal is larger than that of the inner side. That means the planar meridians will experience compression in the wrap-like transfer printing process, which is inevitable in transforming a developable surface into a nondevelopable 3D surface. Take an assumption that the ratio of the arc length at any point to the entire meridian length is equal in the 3D sphere and the 2D pattern, which is

$$\frac{S(y)}{S(\pi R)} = \frac{\theta R}{\pi R} = \frac{\theta}{\pi}$$

Therefore, the planar coordinate (*x*, *y*) of the point *P* can be determined by solving the following equation system

$$\begin{cases} x = R \arctan\left(\sin\left(\frac{y}{R} \right) \tan(\varphi) \right) \\ \frac{S(y)}{S(\pi R)} = \frac{\theta}{\pi} \end{cases}$$

Finally, the planar coordinate (*x*, *y*) of the point *P* can be calculated by spherical coordinate (*R*, *θ*, *φ*), which gives the space mapping relation between the target 3D curve and the corresponding 2D pattern.

Several experiments have been carried out to verify the space mapping relation. Take the spherical parallels and meridians as an example (fig. S10C), for the spherical parallels from θ = 22.5° to θ = 157.5° (the latitude gradient is 22.5°), the corresponding 2D pattern on the petal-like stamp is determined using the space mapping relation (fig. S10D). Similarly, for the spherical meridians from φ = 0° to φ = 330° (the longitude gradient is 30°), the corresponding 2D pattern on the petal-like stamp is determined (fig. S10E). The planar pattern on the petal (fig. S10F) is printed to the sphere surface by wrap-like transfer printing, and the experimental results agree well with the theoretical shape of the parallels and meridians on the sphere (fig. S10G-H).

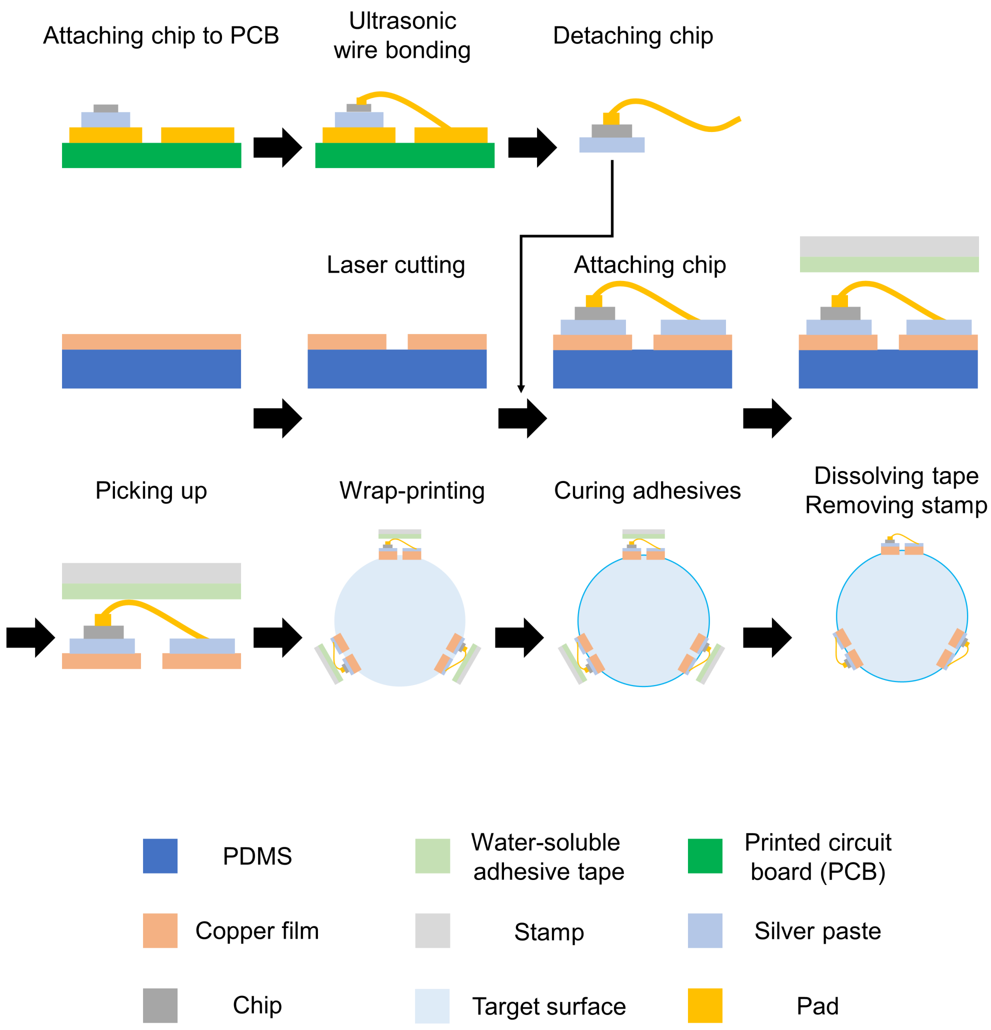

fig. S1. Schematic illustration of the fabrication process of 3D curvy electronics by wrap-like transfer printing. First, a copper foil was bonded to the PDMS by electrostatic bonding. Then, the copper foil was cut into the designed shape by laser cutting. After that, the chips with hanging gold wires were interconnected on the circuit. Next, the planar circuitry was aligned and picked up by the conformal petal-like transfer stamp carefully. After that, the planar circuitry was transfer printed onto a sphere by wrap-like transfer printing. Next, the device was illuminated by ultraviolet exposure to cure the adhesives between the electronics and the spherical surface. Finally, the petal-like stamp was removed by water soaking and the fabrication of 3D curvy electronics was completed.

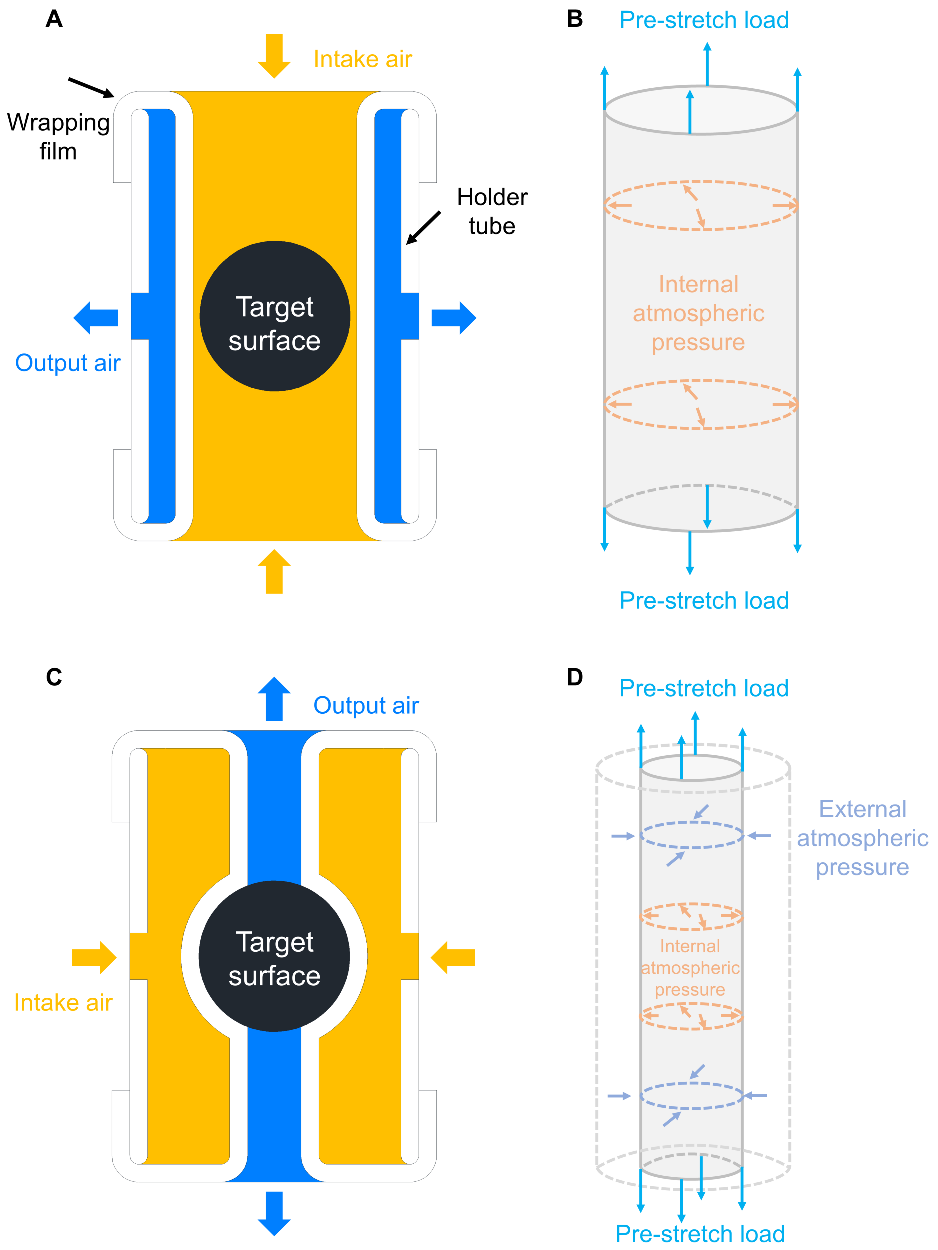

fig. S2. The mechanical equilibrium of the wrapping film. (A) Before wrapping, the air between the holder tube and the wrapping film is extracted out from the side holes of holder tube. (B) Before wrapping, the mechanical equilibrium of wrapping film is governed by internal atmospheric pressure, pre-stretch load, and tensile stress in the wrapping film. (C) After wrapping, the air is blown back to the space between the holder and the wrapping film. (D) After wrapping, the mechanical equilibrium is governed by external atmospheric pressure, internal atmospheric pressure, pre-stretch load, and updated tensile stress in the wrapping film.

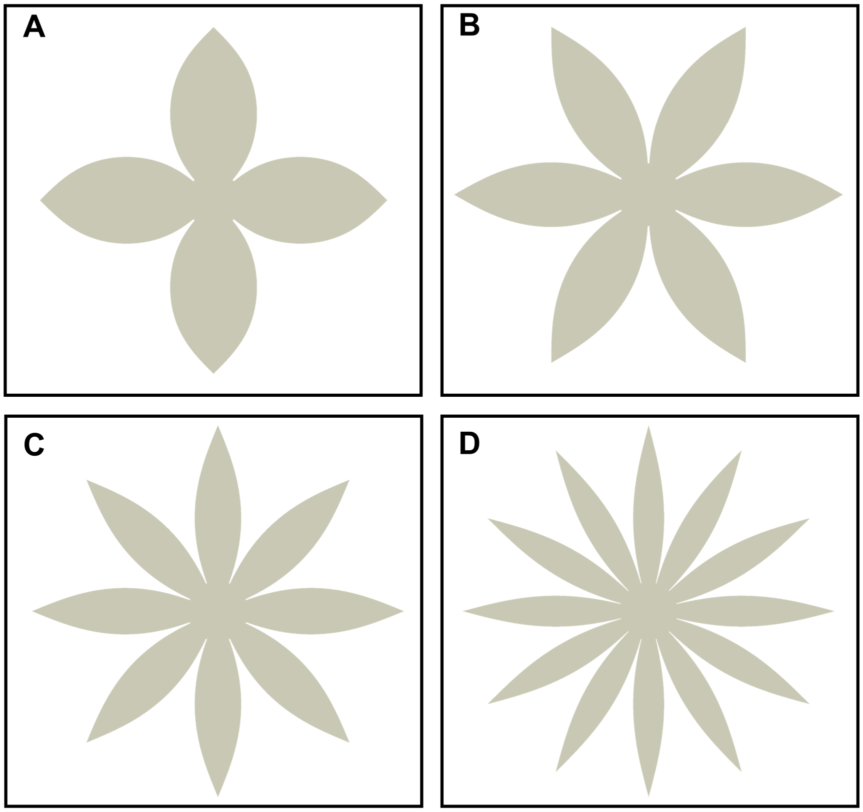

fig. S3. The planar geometry of *k*-petal stamp. (A) $k = 4$. (B) $k = 6$. (C) $k = 8$. (D) $k = 12$.

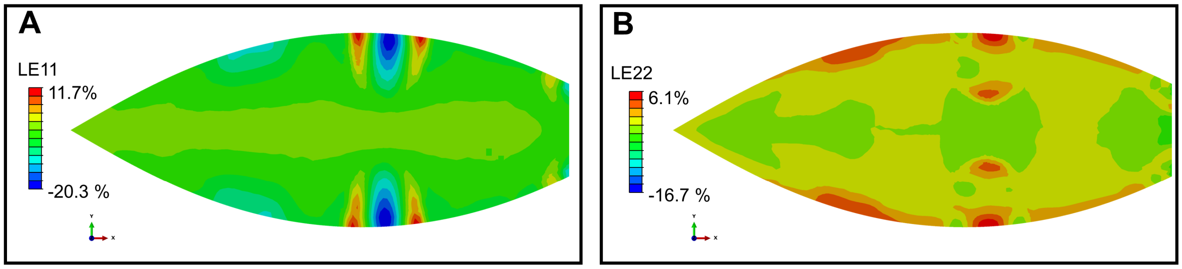

fig. S4. The strain contour of the 6-petal stamp with thickness of 300 μm after wrapping. (A) Normal strain along *x* direction. (B) Normal strain along *y* direction.

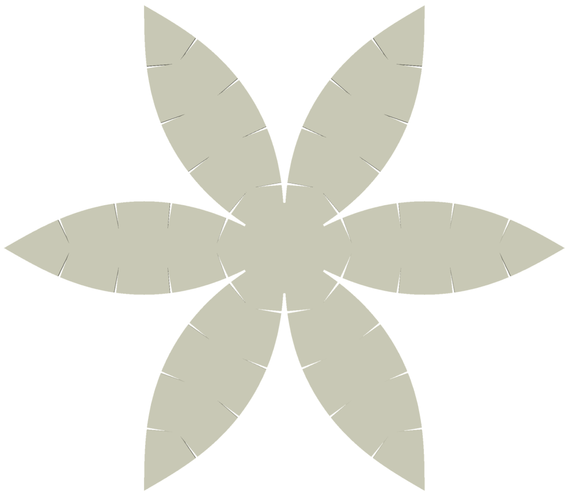

fig. S5. The cutting treatment attempted on the basis of the petal-like design.

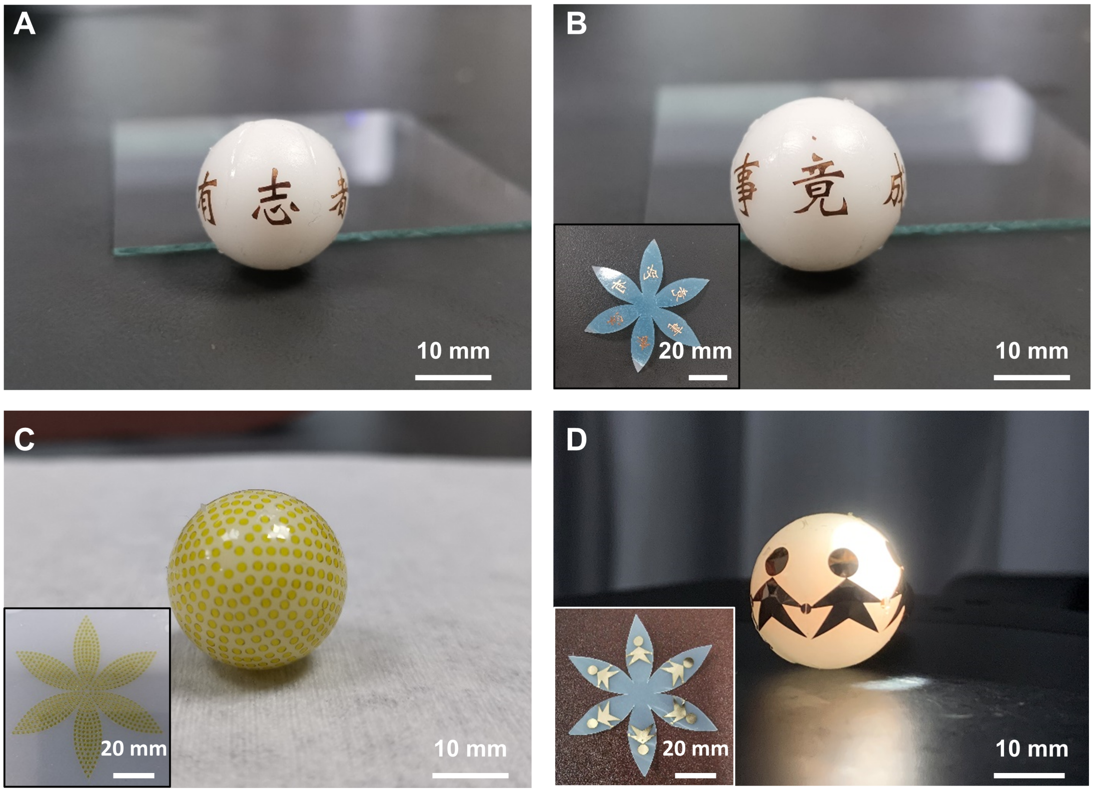

fig. S6. Various pattern integrated on the sphere by wrap-like transfer printing. (A-B) Chinese characters. (C) Round particles. (D) Humanoid pattern. The inset is the corresponding 2D pattern.

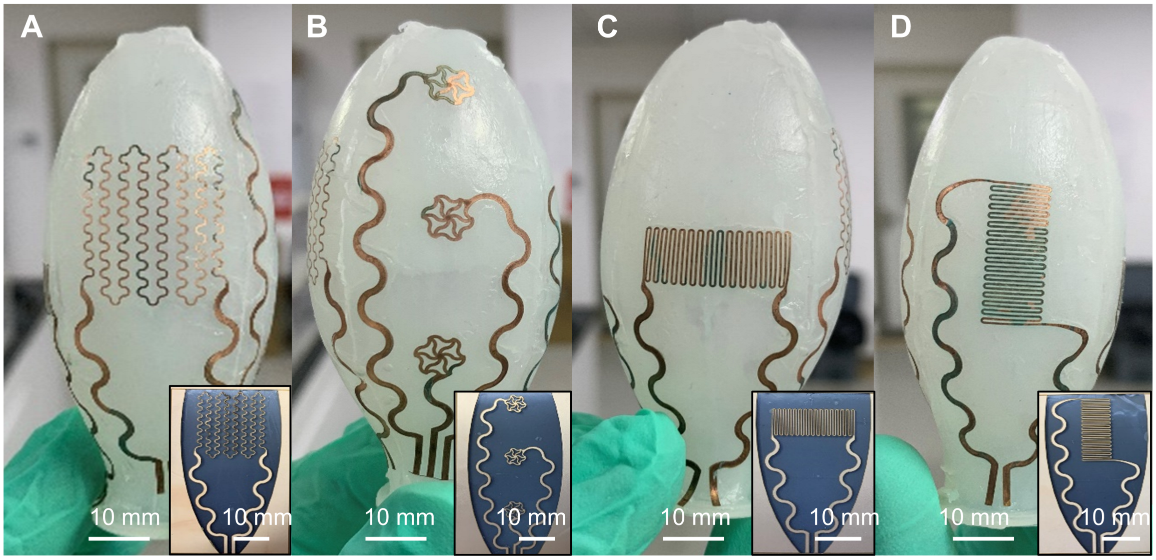

fig. S7. Diverse electrodes integrated on an expandable balloon by wrap-like transfer printing. The inset is the corresponding 2D pattern.

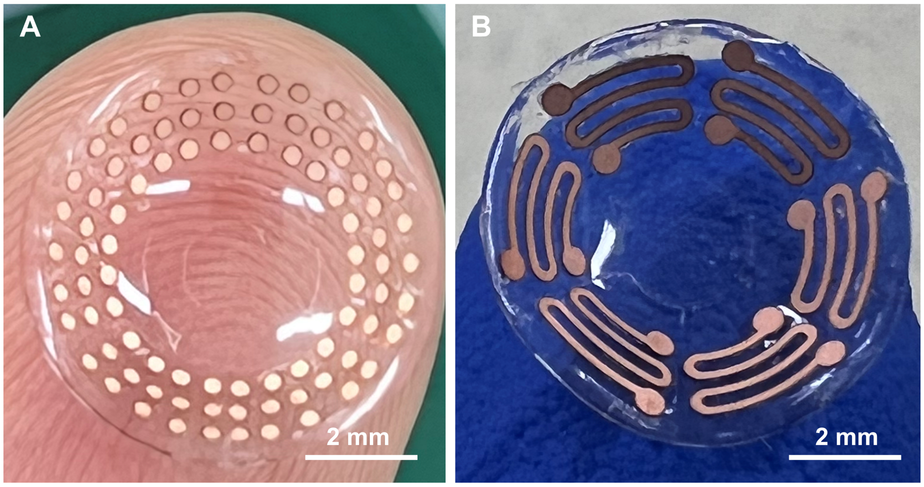

fig. S8. Diverse electrodes integrated on a contact lens by wrap-like transfer printing. (A) Circular dot array and. (B) Serpentine electrodes.

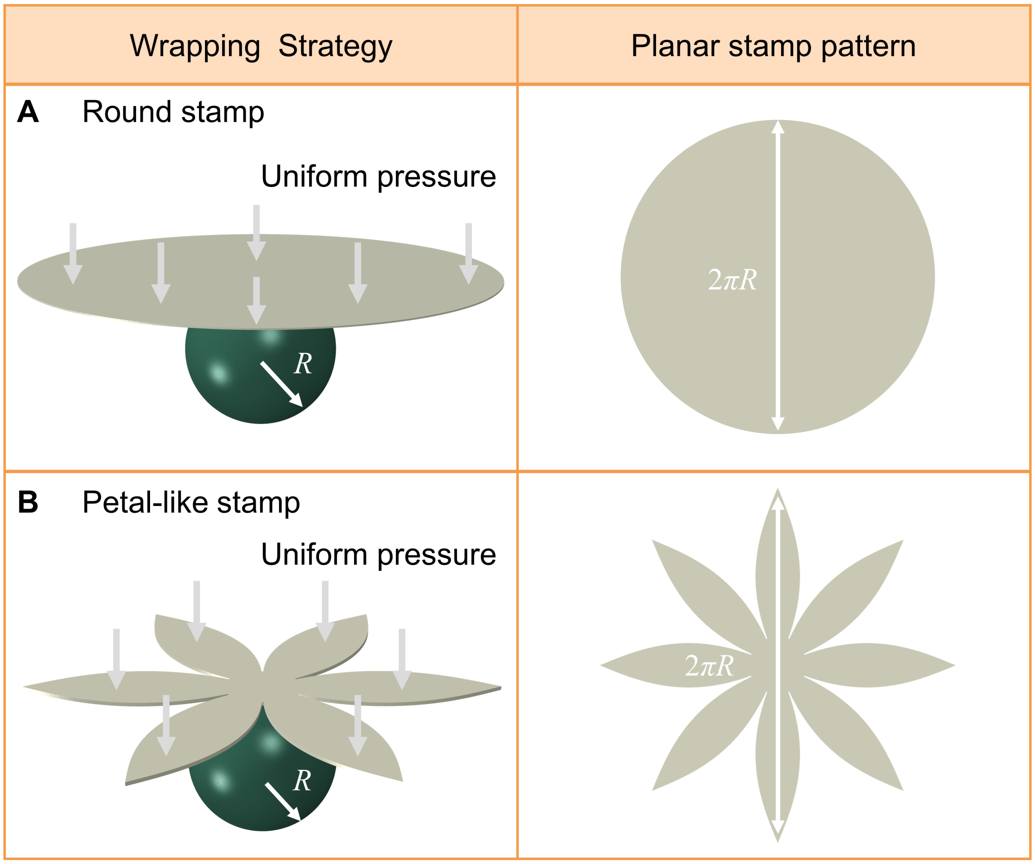

fig. S9. Different wrapping strategies and corresponding planar stamp pattern. (A) Round stamp. (B) Petal-like stamp. Uniform pressure load was applied to the upper surface of the stamp (the pressure direction is perpendicular to the surface) in the wrapping process.

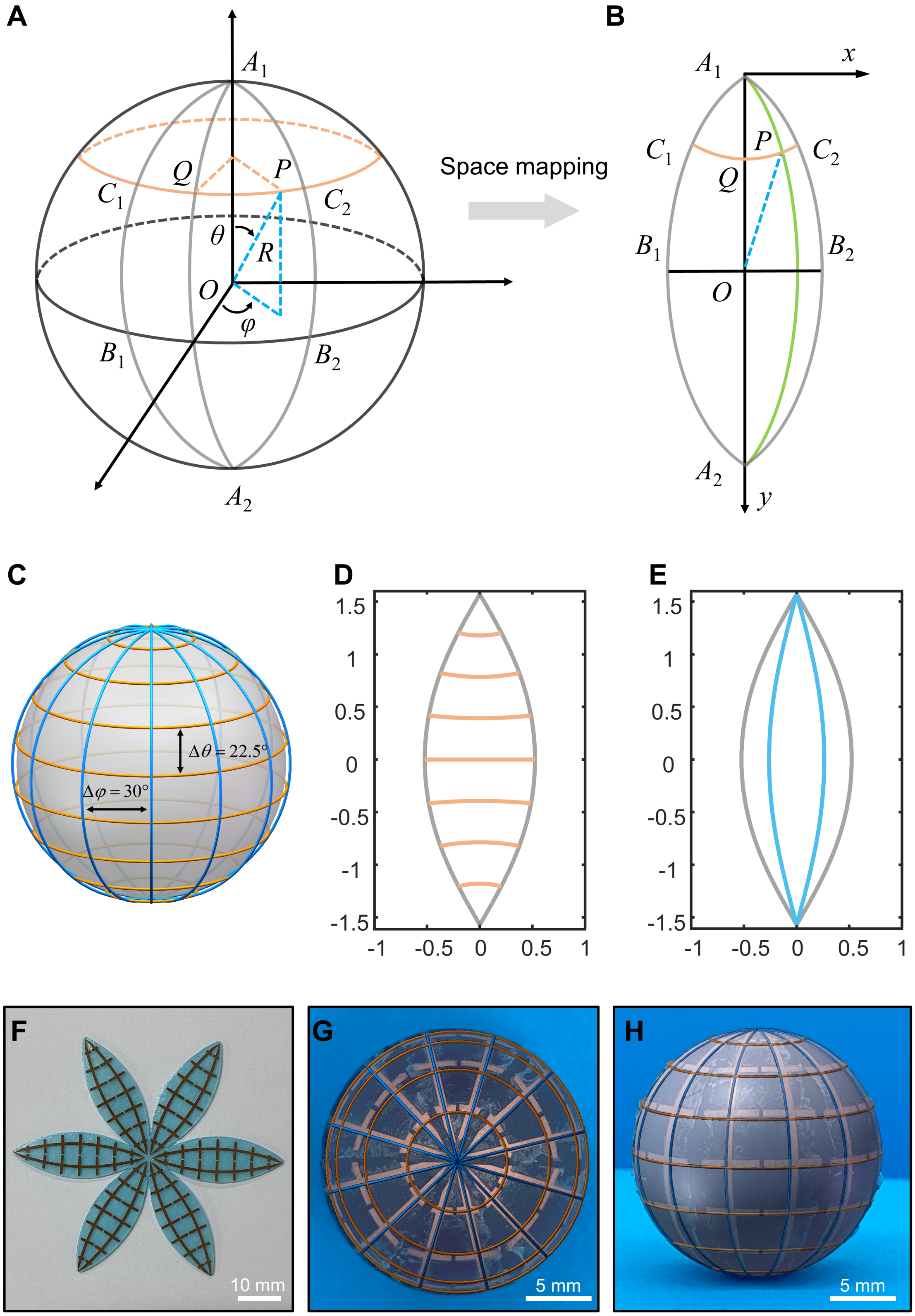

A
B
Space mapping
C
D
E
F
G
H
A_1
A_2
B_1
B_2
C_1
C_2
P
Q
O
R
θ
φ
x
y
$\Delta\theta = 22.5°$
$\Delta\varphi = 30°$
1.5
1
0.5
0
-0.5
-1
-1.5
10 mm
5 mm
5 mm

fig. S10. The space mapping relation between the target 3D curve and the corresponding 2D pattern. (A) The target 3D curve. (B) The corresponding 2D pattern. (C) The spherical parallels and meridians. (D) The corresponding 2D pattern of parallels. (E) The corresponding 2D pattern of meridians. (F) The corresponding 2D pattern on the petal. (G-H) Top view and front view of the transfer printing curves on the sphere (theoretical target curves are marked).

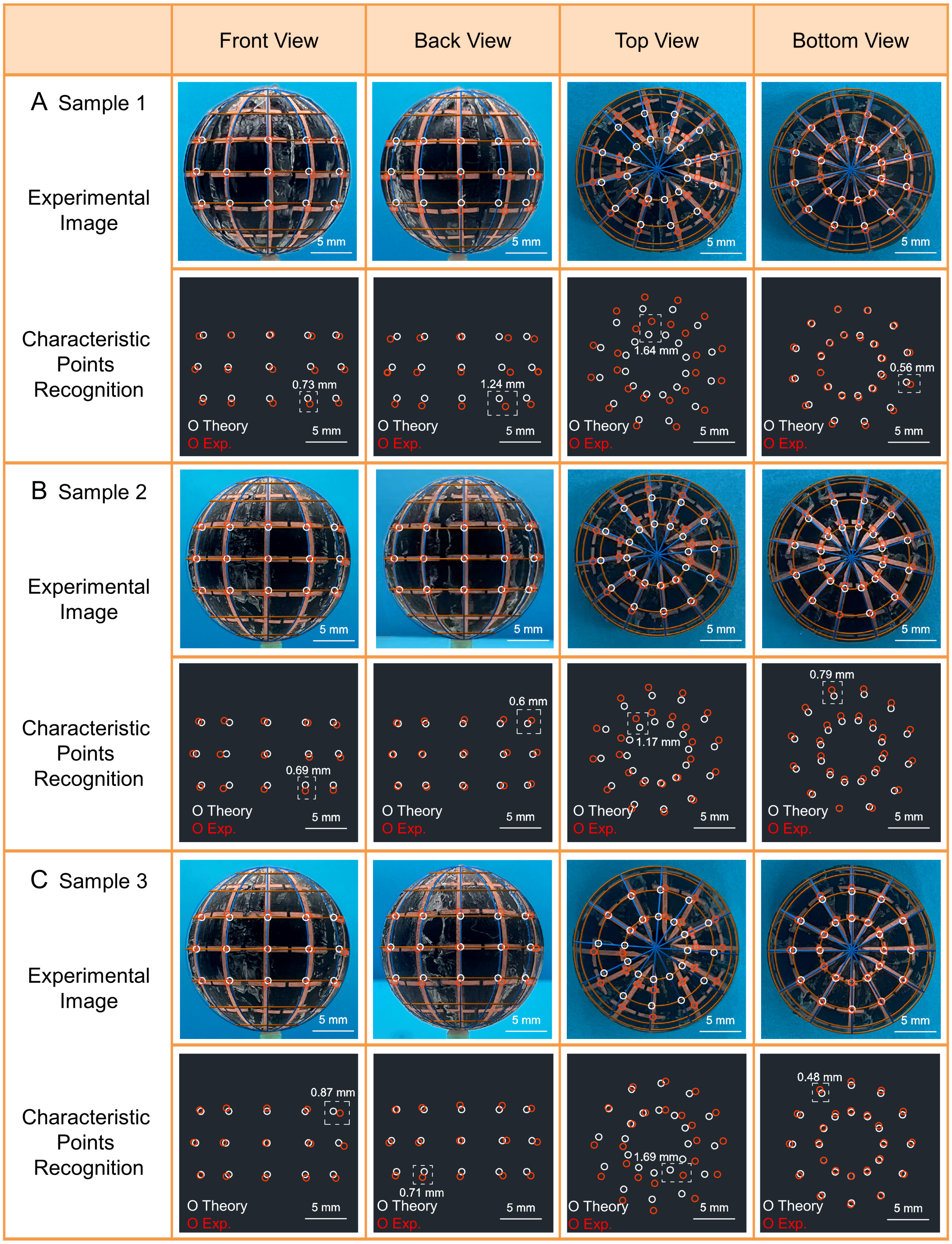

fig. S11. Front view, back view, top view and bottom view of the transfer printing curves on the sphere (theoretical target curves are marked). (A) Experimental image and characteristic points of sample 1. (B) Experimental image and characteristic points of sample 2. (C) Experimental image and characteristic points of sample 3.

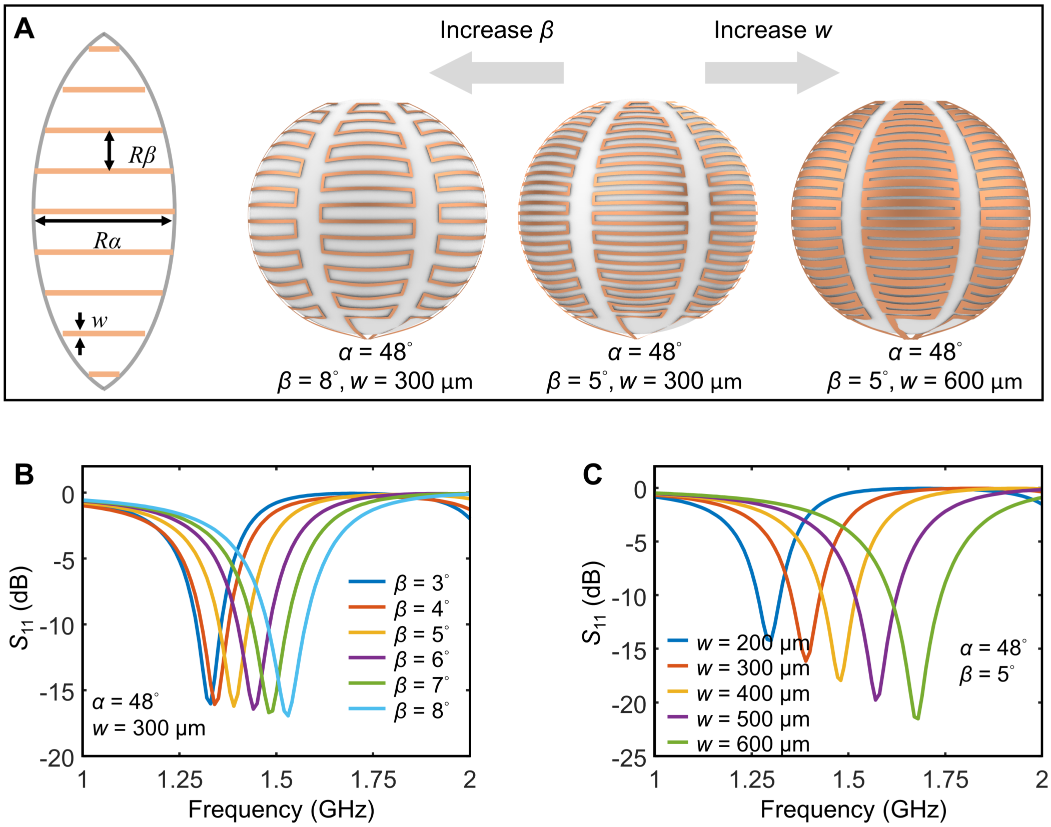

fig. S12. The optimization of design parameters of the spherical meander antenna. (A) The parameter α, β, w of spherical meander antenna. (B) The reflection coefficient S_{11} of the spherical meander antenna with different α (α = 60°, w = 300 μm). (C) The reflection coefficient S_{11} of the spherical meander antenna with different β (α = 48°, β = 5°).

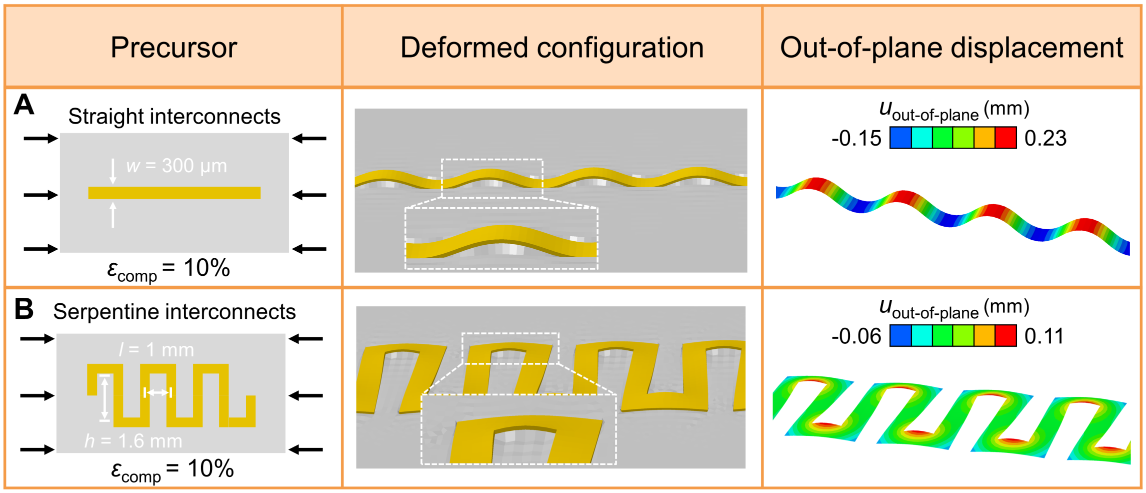

fig. S13. FEA prediction of deformation configuration and out-of-plane displacement of different interconnects design. (A) Straight interconnects (the thickness is 30 μm, the line width is 300 μm). (B) Serpentine interconnects (the thickness is 30 μm, the line width is 300 μm, the serpentine height is 1.6 mm, the serpentine width is 1 mm). The applied compressive strain is 10% for each case.

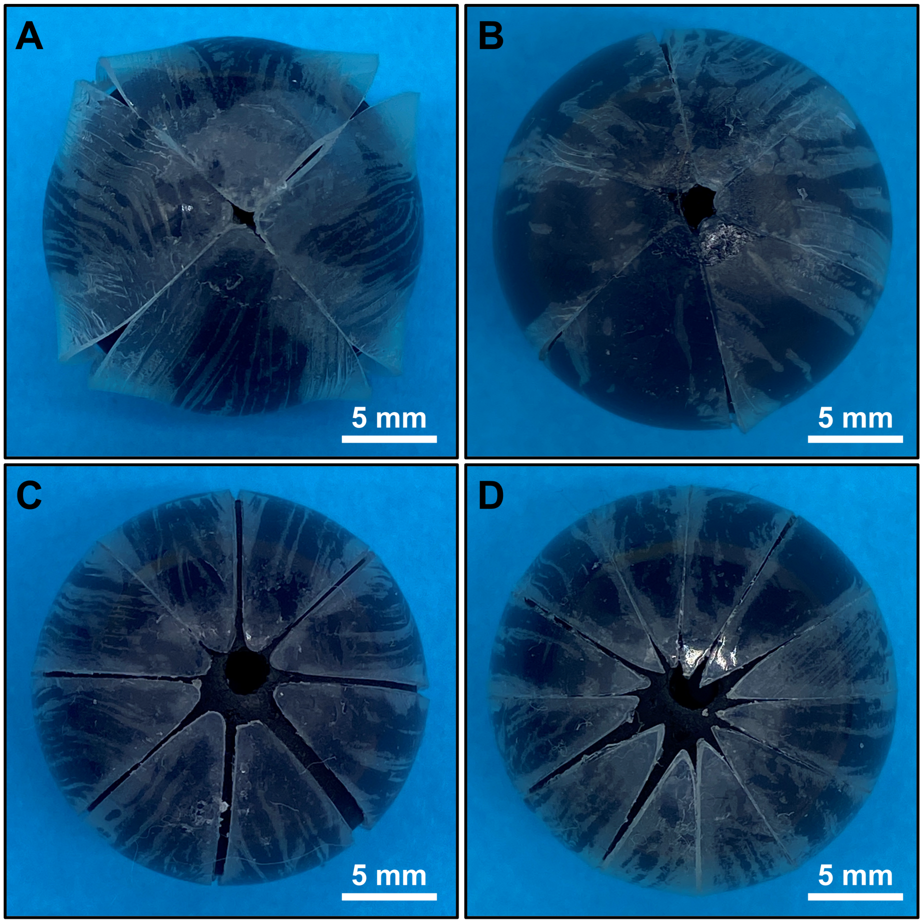

fig. S14. The position of petal tip after wrapping. (A) Petal number is 4. (B) Petal number is 6. (C) Petal number is 8. (D) Petal number is 12.

table S1. Characteristics of methods for building 3D electronic devices.

Method	3D printing	Holographic lithography	Kirigami/ Origami assembly	Direct transfer printing	Transfer printing before shaping	This work
Material compatibility	☆	☆☆☆	☆☆☆	☆☆☆	☆☆☆	☆☆☆
Surface coverage ability	☆☆☆	☆☆☆	☆☆☆	☆	☆	☆☆☆
Cost	☆	☆	☆☆☆	☆☆☆	☆☆☆	☆☆☆
Precision	☆☆☆	☆☆☆	☆	☆☆	☆☆	☆☆
Ref.	26, 27	28	29-32	36, 37	38	

Supplementary Movies:

Movie S1. The wrapping process of the petal-like stamp assisted by the strain recovery of a pre-strained elastic film.

Movie S2. The 360° lighting performance of the spherical LED array.
